# Uncovering the transcriptional response of popcorn (Z*ea mays* L. var. *everta*) under long-term aluminum toxicity

**DOI:** 10.1038/s41598-021-99097-z

**Published:** 2021-10-04

**Authors:** Vitor Batista Pinto, Priscila Gonçalves Ferreira, Pedro Marcus Pereira Vidigal, Tiago Antônio de Oliveira Mendes, Maximiller Dal-Bianco, Jurandir Vieira de Magalhaes, José Marcelo Soriano Viana

**Affiliations:** 1 Departamento de Biologia Geral and Laboratório de Bioquímica Genética de Plantas/BIOAGRO, Universidade Federal de Viçosa, Viçosa, MG 36570-000 Brazil; 2 Laboratório de Biologia Sintética e Modelagem de Sistemas Biológicos, Universidade Federal de Viçosa, Viçosa, MG 36570-000 Brazil; 3 Núcleo de Análise de Biomoléculas (NuBioMol), Centro de Ciências Biológicas, Universidade Federal de Viçosa, Viçosa, MG 36570-000 Brazil; 4 Laboratório de Bioquímica Genética de Plantas/BIOAGRO, Universidade Federal de Viçosa, Viçosa, MG 36570-000 Brazil; 5 Embrapa Milho e Sorgo, Rodovia MG 424 km 65, Sete Lagoas, MG 35701-970 Brazil; 6 Departamento de Biologia Geral, Universidade Federal de Viçosa, Viçosa, MG 36570-000 Brazil

**Keywords:** Plant breeding, Abiotic

## Abstract

To date, the investigation of genes involved in Al resistance has focused mainly on microarrays and short periods of Al exposure. We investigated genes involved in the global response under Al stress by tracking the expression profile of two inbred popcorn lines with different Al sensitivity during 72 h of Al stress. A total of 1003 differentially expressed genes were identified in the Al-sensitive line, and 1751 were identified in the Al-resistant line, of which 273 were shared in both lines. Genes in the category of “response to abiotic stress” were present in both lines, but there was a higher number in the Al-resistant line. Transcription factors, genes involved in fatty acid biosynthesis, and genes involved in cell wall modifications were also detected. In the Al-resistant line, GST6 was identified as one of the key hub genes by co-expression network analysis, and ABC6 may play a role in the downstream regulation of CASP-like 5. In addition, we suggest a class of SWEET transporters that might be involved in the regulation of vacuolar sugar storage and may serve as mechanisms for Al resistance. The results and conclusions expand our understanding of the complex mechanisms involved in Al toxicity and provide a platform for future functional analyses and genomic studies of Al stress in popcorn.

## Introduction

Aluminum (Al) is the third most abundant element in the earth’s crust. In acid soils with pH values at or below 5, the phytotoxic species Al^3+^ is solubilized in soil solution and becomes one of the most important abiotic stresses that limit crop production. Al stress occurs in approximately 30% of the world’s arable soils and in more than 50% of potentially arable land. Of this total, approximately 60% is located in tropical and subtropical regions and negatively impacts the food supply chain. The phytotoxic form Al^3+^ inhibits root growth, thereby altering water and nutrient absorption and consequently reducing plant development^1–3^.

Plants use multiple strategies against Al stress, and two types of mechanisms have been described: (1) Al exclusion, which prevents the entrance of Al into the root apex, and (2) tolerance mechanisms, where Al enters the plant and is detoxified and sequestered^3^. The well-characterized exclusion mechanism is dependent on organic acid (OA) exudation from the root apex^4,5^. Citrate release from the root is an important mechanism against Al stress in maize, which has been identified from the citrate transporter Multidrug and Toxic Compound Extrusion 1 (ZmMATE1)^6^. However, this mechanism is not well correlated with Al resistance, suggesting that other Al resistance mechanisms are operating in the roots of maize^7^. Organic acids such as malate, citrate, and oxalate can chelate Al and attenuate Al toxicity^3^. Al exposure induces malate secretion in wheat^8^, *Arabidopsis*^9^, and rapeseed^10^; citrate secretion in sorghum^11^, barley^12^, rice bean^13^, rice^14^, wheat^15,16^, and common maize^17^; and oxalate secretion in buckwheat^18^, spinach^19^, and tomato^20^.

Maize is widely grown on acid soils throughout the tropics and subtropics, causing yield losses of up to 69%^17,21^. In Brazil, maize cultivation has reached around 4156.6 thousand hectares of planted area as of the 2019/2020 harvest^22^. The cultivation of popcorn (*Zea mays* var. *everta*) is expanding with high demand in Brazil and USA, thus attracting the attention of breeders to obtain populations and hybrids that are adapted to Brazilian conditions^23^. In popcorn, Al toxicity affects root development and causes several types of damage and cell disorganization in the apical region, which compromise plant growth and nutrient uptake^24^. The land requirements and cultivation aspects of popcorn cultivation are similar to those of common maize, so it is essential to obtain genotypes that are tolerant of acid soils and to identify candidate genes for use in popcorn breeding programs.

To our knowledge, no studies have investigated the transcriptome profile of popcorn under Al stress, and it remains unclear what genes and mechanisms are involved in the transcriptional regulation of popcorn under Al stress. The transcriptional response of common maize roots has been tracked using a microarray approach after 1, 2, 6, and 24 h of Al exposure in a hydroponic system^25,26^, and in the early developmental stages, the expression patterns of miRNAs were investigated in maize roots under Al stress^27^. Unlike hydroponic experiments, Mattiello et al.^28^ characterized the transcriptional profile of maize roots during one and three days of growth in soil containing toxic levels of Al using a microarray.

To study the genetic control of Al tolerance in popcorn, Rahim et al.^24^ screened 18 inbred popcorn lines and performed relative root growth (RRG), hematoxylin staining, Al content, scanning electron microscopy, and stereoscopic analyses after seven days of stress treatment (160 μM Al^3+^) to identify inbred lines with Al sensitivity. They classified the 11–60 line as the most Al-sensitive line with the lowest RRG values, the greatest Al accumulation, and intense epidermal degradation in the root tips. They also classified the 11–133 line as an Al-resistant line with the highest RRG value, lowest Al accumulation, and lowest damage to the root apices.

To date, transcriptional expression with RNA sequencing techniques has been used to study Al toxicity tolerance mechanisms in other crops such as sugarcane^29^, buckwheat^30^, and tea plants^31^, allowing the identification of new genes involved in the response to this abiotic stress. The investigation of Al-responsive genes in maize has focused mostly on the early developmental stages and using a microarray approach. We believe that in long-term Al exposure, a robust maintenance mechanism is activated and that several components work at the same time. In this study, we present a high-throughput RNA sequencing method to track the transcriptional response of two contrasting popcorn inbred lines, the Al-resistant 11–133 and Al-tolerant 11–60 lines, to uncover candidate genes related to mechanisms of Al toxicity and tolerance in popcorn.

## Results

**Figure 1:**
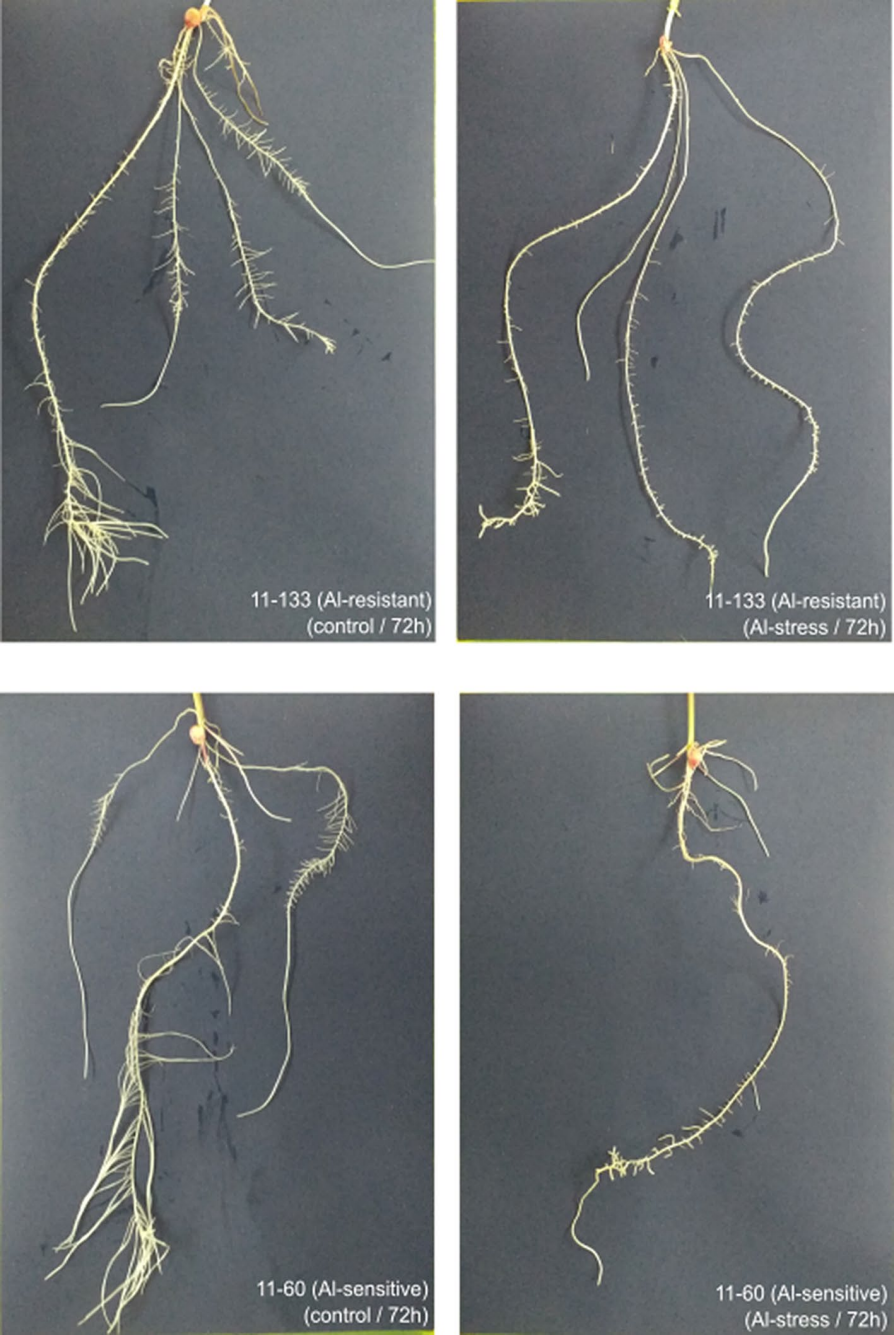
Phenotype of Al-resistant (11–133) and Al-sensitive (11–60) inbred popcorn lines after 72 h in control conditions and Al stress.

We generated a range of ~ 38.5–46.5 million clean reads after a quality process for each sample and obtained around 52.87% GC content. An average of 80.10% of the reads were mapped, and from this total, only 6.33% presented multiple alignments with the B73 reference genome using the default parameters (Supplementary Table S1). We performed a Principal Component Analysis (PCA) to compare the differentially expressed genes (DEGs), which revealed that the DEGs from the control and + Al treatments were clustered together for both lines, but the lines were grouped into separated clusters, showing differences in the genetic background between both inbred lines (Supplementary Fig. S1). After 72 h of stress treatment, the inbred lines with different Al sensitivity presented visual phenotypic differences. The Al-sensitive line demonstrated changes in root development and a decreased number of roots (Fig. 1).

**Figure 2:**
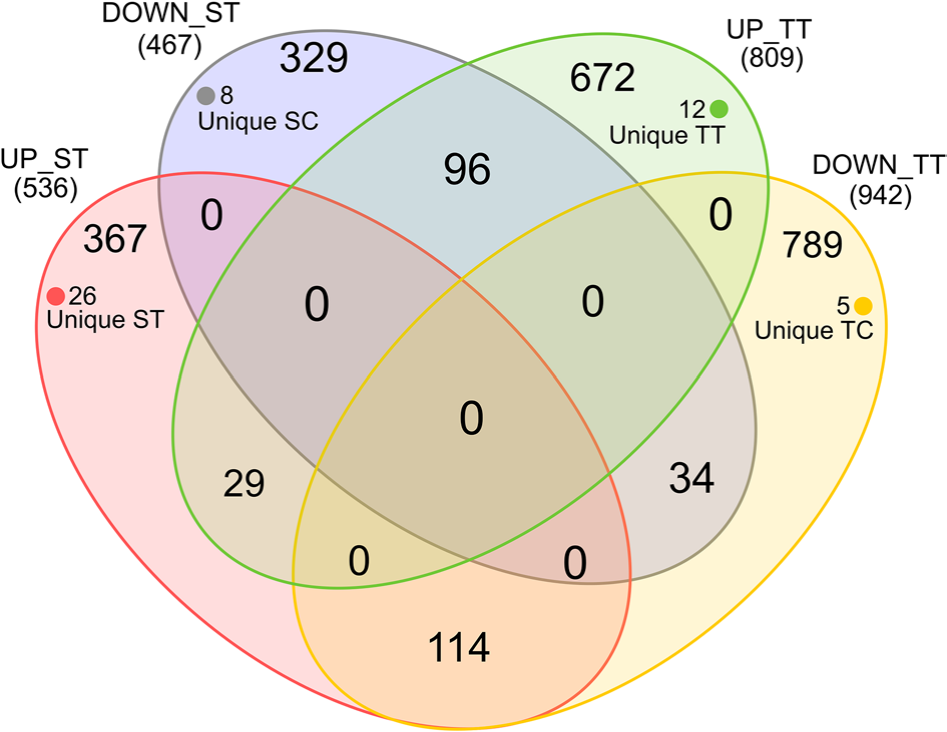
Venn diagram of DEGs under Al stress (*UP_TT* up-regulated genes in Al resistant line, *DOWN_TT* down-regulated genes in Al resistant line, *UP_ST* up-regulated genes expressed in Al-sensitive line, *DOWN_ST* down-regulated genes expressed in Al-sensitive line).

**Figure 3:**
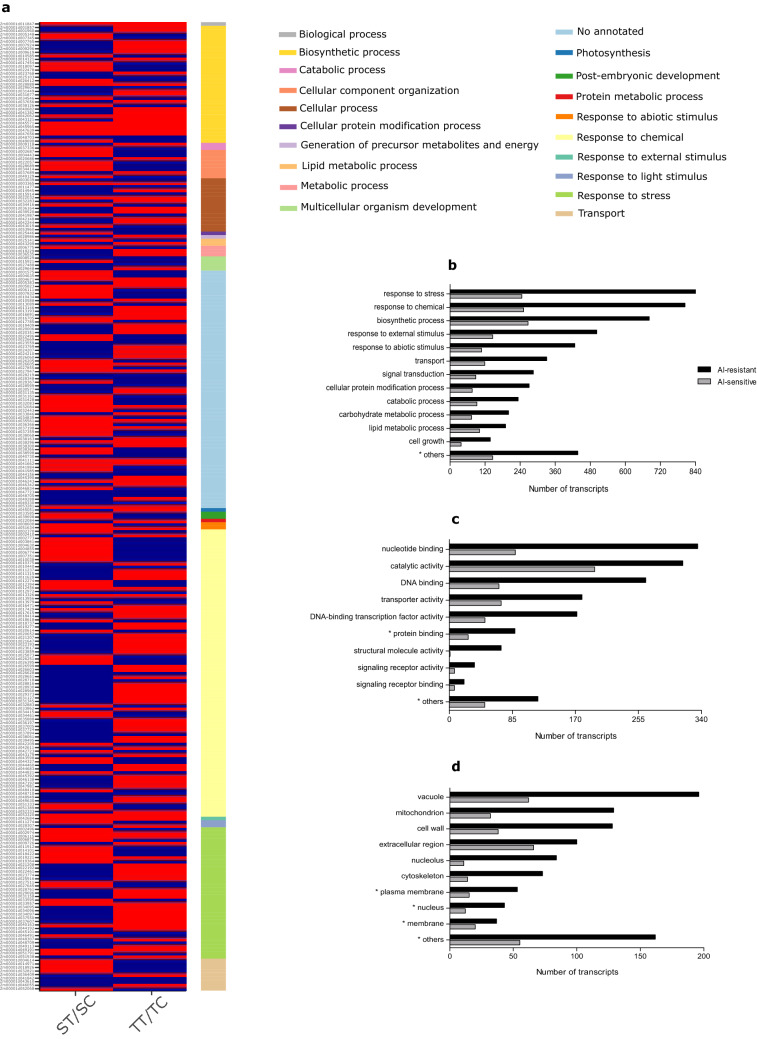
Gene Ontology (GO) enrichment of DEGs. (**a**) Heatmap of the differentially expressed genes shared in both inbred lines—ST/SC: contrast between Al-sensitive line under treatment/control conditions; TT/TC: contrast between Al-resistant line under treatment/control conditions. (**b**) Biological process. (**c**) Molecular function. (**d**) Cellular component. *The numbers of genes were divided by 10. Heatmap was generated in R version 3.6.2 (https://www.r-project.org/).

We detected a total of 1003 DEGs in the Al-sensitive line (467 down-regulated and 536 up-regulated) along with 1751 DEGs in the Al resistant line (942 down-regulated and 809 up-regulated) at a False Discovery Rate (FDR) of q < 0.01 (Fig. 2; Supplementary Table S2). The different numbers of DEGs in both inbred lines show that the Al-resistant line has a broader response to Al stress than the Al-sensitive line, regulating multiple pathways against Al damage. Furthermore, we found 273 common DEGs in both inbred lines under Al stress (Fig. 3a; Supplementary Table S3). The functional analysis indicated that the broadest range of these genes is involved in “response to chemicals,” followed by “response to stress” and “biological process” (Fig. 3a). There were 114 DEGs that were up-regulated in the Al-sensitive line and presented down-regulation in the Al resistant line. There were also 96 genes that had the opposite behavior, showing down-regulation in the Al-sensitive line and up-regulation in the Al resistant line (Fig. 3a; Supplementary Table S2).

A Gene Ontology (GO) analysis was performed to categorize DEGs into different groups. For the Al-resistant line, the majority of the DEGs were classified as the “response to stress” category, but in the Al-sensitive line was highly enriched in the “biosynthetic process” category. In the biological process category, several terms involved in stress response were much more enriched in the Al-resistant line than in the Al-sensitive line (Fig. 3b). A total of 427 DEGs were clustered in the “response to abiotic stimulus” in the Al-resistant line, which is almost 4 times more than in the Al-sensitive line. This was also observed in the terms “signal transduction” and “cell growth” (Fig. 3b). The terms “protein binding” and “nucleotide binding” in the molecular function category (Fig. 3c) and “plasma membrane”, “nucleus”, and “membrane” in the cellular component category (Fig. 3d) were highly enriched in both inbred lines.

Differentially expressed transporters were also found in both inbred lines (Tables 1 and 2), including Natural resistance-associated macrophage protein (Nramp), aquaporins, Sugars Will Eventually be Exported Transporter (SWEET), Al-activated malate transporter (ALMT), MATE, and ABC transporters. The majority of transporters regulated in both inbred lines were ABC transporters from the G and B families. We detected an increase of regulation of the gene ABC G member 29 (Zm00001d043598) in the Al-resistant line (Table 2), while the same gene presented an opposite regulation profile in the Al-sensitive line (Table 1).

**Table 1:** Aluminum responsive transporters related to Al-sensitive inbred line.

Class	Gene ID	Transporter	Fold change
ABC transporter	Zm00001d043598	ABC transporter G family member 29	− 2.438
	Zm00001d032279	ABC2 homolog 15	− 1.448
	Zm00001d025703	ABC transporter B family member 15	− 1.376
	Zm00001d011315	ABC transporter G family member 40	1.804
	Zm00001d026041	ABC transporter C family member 9	1.788
	Zm00001d021647	ABC transporter G family member 34	1.068
SWEET	Zm00001d050577	SWEET 15a	2.208
	Zm00001d015914	SWEET 4b	2.195
	Zm00001d010440	SWEET 3a	1.033
Nramp	Zm00001d019327	Metal transporter Nramp6	− 1.095
	Zm00001d014391	*Nrat1*	1.783
ALMT	Zm00001d026102	Aluminum-activated malate transporter 10	1.983

**Table 2:** Aluminum responsive transporters related to Al-resistant line inbred line.

Class	Gene ID	Transporter	Fold change
ABC transporter	Zm00001d011315	ABC transporter G family member 40	− 2.855
	Zm00001d044442	ABC transporter G family member 40	− 2.110
	Zm00001d028870	ABC transporter family protein	− 1.661
	Zm00001d046225	ABC transporter family protein	− 1.489
	Zm00001d046226	*mrpa1* (ABC) transporter	− 1.363
	Zm00001d021647	ABC transporter G family member 34	− 1.328
	Zm00001d004361	ABC transporter C family member 4	− 1.005
	Zm00001d043598	ABC transporter G family member 29	2.598
	Zm00001d024600	ABC transporter B family member 19	2.564
	Zm00001d043766	ABC transporter B family member 9	1.813
	Zm00001d036986	ABC transporter G family member 29	1.398
	Zm00001d044564	ABC transporter B family member 9	1.189
	Zm00001d042953	ABC transporter G family member 6	1.135
SWEET	Zm00001d011299	SWEET 6b	− 3.553
	Zm00001d029135	SWEET 12a	3.418
	Zm00001d023673	SWEET 13b	2.714
	Zm00001d015905	SWEET 4a	1.505
	Zm00001d041067	SWEET 13c	1.429
	Zm00001d010440	SWEET 3a	1.059
	Zm00001d015914	SWEET 4b	1.011
Aquaporin	Zm00001d022608	Aquaporin PIP2-2	− 2.072
	Zm00001d048520	Aquaporin TIP3.1	− 1.813
	Zm00001d005410	Aquaporin PIP2-2	1.543
	Zm00001d014285	Aquaporin PIP2-2	1.186
ALMT	Zm00001d046029	Aluminum-activated malate transporter 10	1.072
Heavy metal transporters	Zm00001d002496	Heavy metal transport/detoxification superfamily protein	1.679
	Zm00001d026298	Putative heavy metal transport/detoxification protein	1.295
MATE	Zm00001d009494	Putative MATE efflux family protein	− 2.962

Nramp aluminum transporter 1 (Nrat1, Zm00001d014391) was up-regulated, while Nramp6 (Zm00001d019327) was down-regulated in the Al-sensitive line (Table 1). We also found four genes encoding aquaporin proteins in only the Al-resistant line (Table 2). Two Aquaporin PIP2-2 genes (Zm00001d005410 and Zm00001d014285) were up-regulated, while Aquaporin TIP3-1 (Zm00001d048520) and an Aquaporin PIP-2-2 ortholog (Zm00001d022608) were down-regulated. We also identified seven SWEET transporters, and all of them were up-regulated in both inbred lines except SWEET 6b, which was down-regulated in the Al-resistant line (Table 2).

Genes related to reactive oxygen species (ROS) protection were present in both inbred lines, but there were almost 2 times more in the Al-resistant line than in the Al-sensitive line. We detected up-regulated genes with glutathione S-transferase (GST) activity (Table 3), and the majority were in the Al-resistant line. Additionally, genes playing a role in ROS scavenging, such as peroxidase (POD), catalase (CAT), reductase (RE), and cytochrome P450, were detected in both inbred lines (Supplementary Table S3).

**Table 3:** Glutathione S-transferases identified in RNA sequencing of Al-sensitive and Al-resistant inbred lines under 72 h of Al stress.

Gene ID	GST protein	Fold change
**Al-sensitive**		
Zm00001d029706	*gst39*	2.363
Zm00001d029696	*gst34*	1.477
Zm00001d029702	Glutathione S-transferase U16	− 1.212
Zm00001d048558	gst25	1.298
**Al-resistant**		
Zm00001d048353	*gst13*	2.034
Zm00001d029707	*gst38*	1.862
Zm00001d042104	*gst7*	1.664
Zm00001d027539	*gst11*	1.305
Zm00001d029699	*gst42*	1.266
Zm00001d029801	*gst14*	1.262
Zm00001d048354	*gst9*	1.196
Zm00001d027540	*gst12*	1.080
Zm00001d038192	*gst41*	1.073
Zm00001d028692	Glutathione S-transferase L2 chloroplastic	1.070
Zm00001d047765	Glutathione S-transferase L2 chloroplastic	1.027
Zm00001d029696	*gst34*	− 1.153

Interactive Pathways Explorer analysis was performed using the KEGG Orthology (KO) database, which revealed that various pathway maps in the Al-resistant line were highly modified under aluminum stress in comparison with the Al-sensitive line (Supplementary Figs. S2 and S3). Curiously, in the Al-resistant line, we detected several genes in the “lipid metabolism” pathway (Supplementary Fig. S2), in contrast with just a few genes in the Al-sensitive line (Supplementary Fig. S3). Several transcription factors (TFs) were detected among our differentially expressed genes. There were 32 down-regulated and 23 up-regulated TFs in the Al-sensitive line, and the Al-resistant line presented 89 down-regulated and 38 up-regulated TFs (Supplementary Table S4). Among these, we found TFs that belonged to the families AP2/EREBP, MYB, bHLH, and WRKY. We also found TFs that are exclusively overexpressed in the Al-resistant line, including ZF-HD, ARF, and E2F/DP families.

From the list of DEGs, nine genes were selected for experimental validation by RT-qPCR. The selected genes are related to abiotic stress: transcription factor HY5-like (HY5), pectin methyltransferase (PME), SWEET 12a, ALMT 10, brassinosteroid catabolism 2 (BRAS), glutathione S-transferase U16 (GST), MYB DNA-binding (MYB), SNF1-related protein kinase regulatory subunit beta-1 (SNF 1), and xyloglucan endotransglucosylase/hydrolase protein 21 (XET). The biological validation was confirmed by RT-qPCR and comparison to RNA-seq data (Supplementary Fig. S4).

## Discussion

Aluminum toxicity is one of the main factors limiting crop cultivation in acidic soils. For more than 50 years, breeders have explored genetic diversity to improve Al resistance in several crops, especially in tropical breeding programs. Some critical Al toxicity events are initiated at the transcriptional, biochemical, and physiological levels. To date, several Al-tolerance mechanisms have been described, but much more is needed to uncover the complex response of Al stress. To date, no study has investigated the global differential responses of resistance to aluminum in popcorn plants. In the current study, a high-throughput RNA sequencing approach was used to measure the transcriptome changes in popcorn (*Zea mays* var. *everta*) roots under a long period (72 h) of Al exposure.

Al toxicity occurs when Al comes into contact with the cell walls, plasma membranes, and cytoplasm of apical root cells^32^. In the cell walls, expansins modify the cellulose and non-cellulosic components, thereby loosening and modifying the plant cell walls during growth and adaptation to biotic and abiotic stress^33^. Recently, the expansin *HvEXPA1* was found to be inducible in barley roots under Al stress and to participate in root-cell elongation and regulation of the loosening of the root cell walls^34^. However, the relationship between expansins (EXP) and Al stress is still poorly understood. In our study, 10 EXPs were detected in the Al-resistant line (Supplementary Table S2). Two EXPA11s (Zm00001d048418 and Zm00001d032883) were up-regulated in the Al-resistant line but down-regulated in the Al-sensitive line, suggesting that these specific EXPs could play an important role in the cell-wall modification under Al stress.

**Figure 4:**
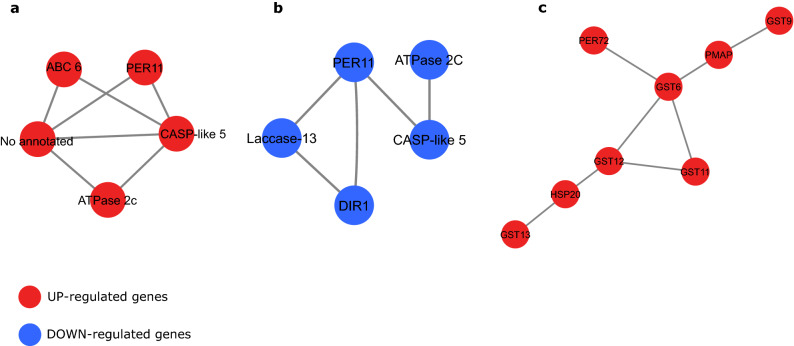
Co-expression network of selected DEGs under Al stress. The first degree of interaction was retrieved from STRING (version 10.5) using a minimum required interaction score of 0.7, and the network analysis performed in Cytoscape (version 3.7.1).

Besides genes involved in cell wall modification, genes encoding membrane transporters are necessary for Al tolerance^35^. Transporters are some of the most important components in plant Al resistance and play a role in the plasma membrane and tonoplast by participating in exclusion and tolerance mechanisms^3^. The ABC transporter families found in both inbred lines (Tables 1, 2) suggest that these transporters work together with other detoxifying systems to increase the tolerance response in the Al-resistant line. In the co-expression network, the *ABC6* transporter (Zm00001d042953) interacts with the protein Casparian strip membrane domain (CASP)-like 5 (Zm00001d010038) (Fig. 4a).

Unlike the Al-resistant line, the same CASP protein was down-regulated, while the *ABC6* was not detected in the Al-sensitive line’s co-expression network (Fig. 4b). CASPs are involved in the formation and location of Casparian strips, nutrient uptake, and stress resistance^36^. Its expression is increased in cotton during cadmium stress^37^ and in Tamba black soybean^38^ during Al stress, thus affecting plant root growth. This interaction suggests the importance of *ABC6* in the downstream regulation of CASP-like 5 under Al stress in popcorn roots.

Nrat1 is a specific Al transporter identified in rice that uptakes Al into cells for sequestration to vacuoles and is required for the initial steps of internal Al detoxification^39^. The best candidate gene as a homolog of OsNrat1, Zm00001d014391, was up-regulated in the Al-sensitive line. Although our Al-sensitive inbred line can respond with multiple mechanisms against the absorption of Al ion in popcorn roots, we propose that this is not sufficient to support this stress due to the metabolic unbalance caused by the Al toxicity conditions. In addition, aquaporins are a group of highly conserved membrane proteins that facilitate water transport across biological membranes^40^. Our observations suggest that tonoplast aquaporins are likely involved in the Al-tolerance response, but it remains unclear whether Aquaporin PIP proteins play an important role in response to Al stress in popcorn.

Cellular efflux of sugar plays an important role in the maintenance of sugar efflux in phloem loading, nectar secretion, and maternal efflux for filial tissue development^41^. Plants need to maintain rigid regulation in the storage and transport of vacuolar sugar to deal with adverse environmental conditions^42^. The role of SWEET in Al-tolerance responses remains unclear, but it plays an important role in adverse conditions, such as salt, osmotic, oxidative, and cold stress, as well as water deficit conditions in *Arabidopsis*^43–45^. The present work demonstrates a broad up-regulation of SWEET transporters under Al stress (Table 2). Thus, we suggest that these SWEET transporters might play a role in maintaining the tight regulation of vacuolar sugar storage and conferring greater flexibility to adapt to the Al-stress environment.

Membrane transport proteins generally mediate the exudation of organic acids in response to Al stress^35^. To model the temporal process featuring the response of the intracellular gene expression profile upon Al stress, the Interactive Pathways Explorer was applied to visualize and customize the various pathway maps using the KO database. In the tricarboxylic acid cycle, down-regulated genes were detected in the via from isocitrate, and up-regulated genes were detected in the via from oxaloacetate in the Al-resistant line (Supplementary Fig. S2). The organization of carboxylic acid metabolism in plants is highly dependent on the metabolic and physiological demands of the cell^46^, and the Al-stress conditions might induce this non-cyclic flux in Al-resistant line. We also observed up-regulated genes involved in the glyoxylate cycle in the Al-resistant line (Supplementary Fig. S2). To our knowledge, no evidence has been presented demonstrating glyoxylate-cycle changes in plants under Al stress, and this hypothesis needs further investigation.

Lipids are the major component of cell membranes, and their composition changes are widely found under various abiotic stresses^47,48^. Up-regulated genes were involved in lipid metabolism in the Al-resistant line (Supplementary Fig. S2), while these genes were mostly down-regulated or not detected in the Al-sensitive line (Supplementary Fig. S3). Abiotic stress induces changes in the fatty acid composition of plant membrane lipids due to the ability to adjust membrane lipid fluidity by changing the level of unsaturated fatty acids^49^. In wheat roots, increased expression of lipid transfer proteins enhanced cutin layer thickness in an Al-tolerant near isogenic line under 3 and 7 days of Al exposure, thus protecting root cells from Al damage^50^. In the Al-resistant line, up-regulated genes are involved in cutin, suberin, and wax biosynthesis, and down-regulated genes are involved in the fatty acid degradation pathway in the Al-resistant line (Supplementary Fig. S2). These results indicate that fatty acids might contribute to the membrane integrity of the Al-resistant inbred line under Al stress.

In several organelles, such as mitochondria, chloroplasts, and peroxisomes, Al stimulates the emergence of ROS, leading to oxidative damage and consequent cell toxicity^35,51^. Several DEGs involved in the antioxidant system and ROS scavenging were identified in our work (Supplementary Table S3). These data are in agreement with those from Mattiello et al.^28^, who detected an up-regulation response of several ROS-related genes in maize growing in acid soils. They suggested that this mechanism acts before the oxidative stress occurs. However, they did not detect the induction of superoxide dismutase (SOD) after 72 h of Al exposure, and the same was observed in our study. Testing two contrasting maize lines under different concentrations of aluminum ions, Giannakoula et al.^52^ showed that the anionic POD isoforms and SOD isoforms increased with increasing Al stress in the tolerant line. In the same way, the CAT enzyme acts as an auxiliary antioxidant that works selectively with either SOD or POD during the peroxidation caused by Al stress as a major enzyme responsible for root growth^53^.

The GSTs are also involved in Al-toxicity response in maize. Xu et al.^25^ identified an increase of GST expression in roots, resulting in a strong Al tolerance in maize. In the same way, Cançado et al.^54^ indicate that a GST may play a role in Al stress alleviation in maize roots. Consistently with these results, GSTs were mostly up-regulated in the Al-resistant line (Table 3) in our RNA-seq data. In the co-expression network analysis, significant patterns of protein–protein interaction were identified between several GSTs, among which *GST6* (Zm00001d027541) interacts with a high number of proteins in the Al-resistant line (Fig. 4c). The GSTs also interact with other important components involved in the abiotic stress response, such as PER72 (Zm00001d009373), heat shock protein 20 (HSP20, Zm00001d030346), and plasma membrane-associated protein (PMAP, Zm00001d009932), thus demonstrating a central role of GSTs in the Al-stress response.

Several classes of TFs were identified in our work. Members of MYB, bHLH, AP2/EREBP, WRKY, and NAC families have been identified to be differentially expressed under Al toxicity in different species in previous work^30,55^. In common maize, a number of TFs were detected in hydroponic^25,26^ and acid soil experiments^28^. In addition, miRNA module-involving TFs such as NAC and MYB appear to play a role in the regulation of crown and seminal root development^27^. TFs belonging to these families were found with different expression regulation in both inbred lines (Supplementary Table S4) and may be involved in the downstream regulation of the expression of genes responsive to Al toxicity.

**Figure 5:**
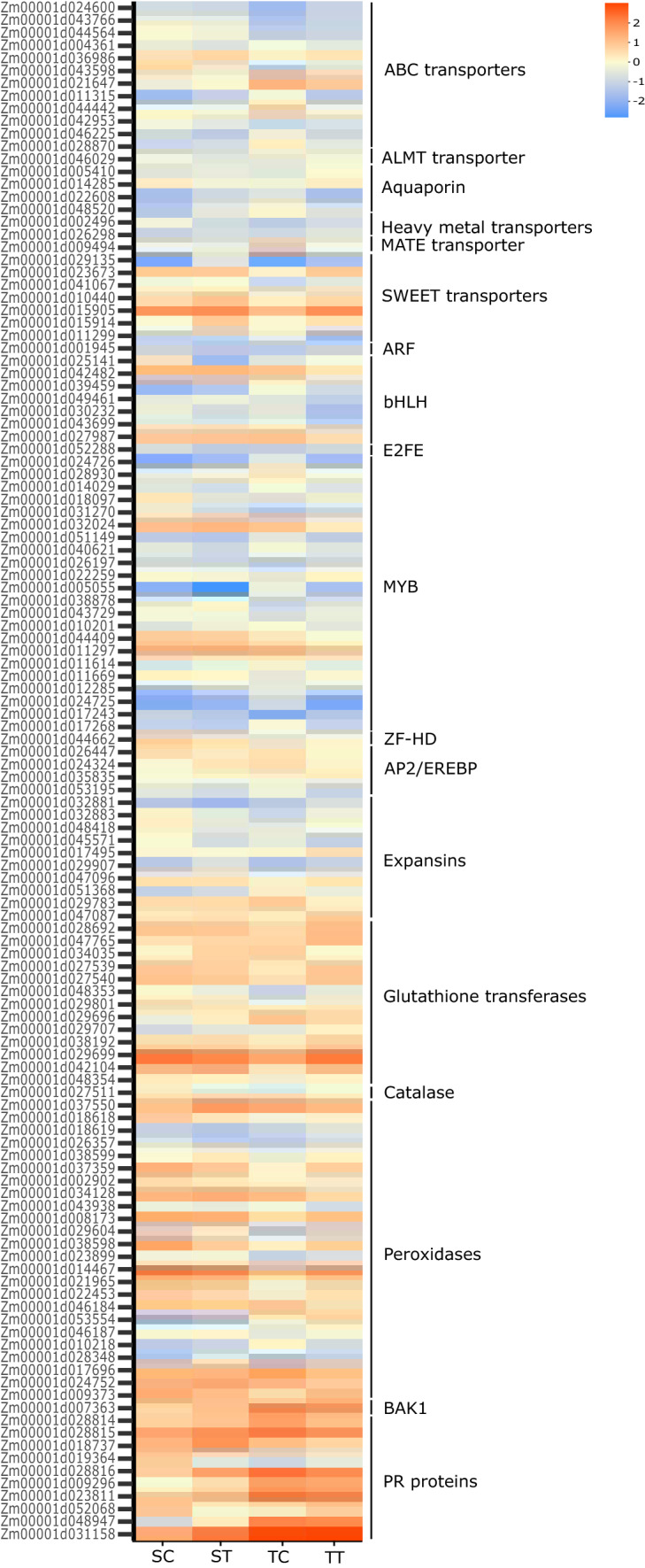
Heatmap of UPGMA clustering of selected candidate genes differentially expressed in Al-resistant inbred line. *SC* Al-sensitive control, *ST* Al-sensitive treatment, *TC* Al-resistant control, *TT* Al-resistant treatment. Heatmap was generated in R version 3.6.2 (https://www.r-project.org/).

Finally, we selected a list of candidate genes involved in the global response to Al toxicity in popcorn (Fig. 5), which opens up an opportunity for the development of molecular markers in popcorn breeding programs. The classes of transporters, EXPs, genes related to ROS protection, hormones signaling, pathogenesis-related (PR) proteins, and TFs were significantly involved in the Al response in popcorn roots. These genes increase our understanding of responses to Al toxicity. Beyond that, SWEET transporters involved in Al stress may act in the regulation of vacuolar sugar storage under Al toxicity. The results open new avenues and could further help us to understand the mechanisms of Al toxicity and tolerance that are regulated at the transcriptional level in long-term exposure of popcorn roots.

## Methods

### Plant materials

Seeds from two contrasting inbred popcorn lines developed by the Popcorn Breeding Program of the Universidade Federal de Viçosa were used in this study: 11–133 (Al-resistant) and 11–60 (Al-sensitive). These genotypes were selected based on a previous study to screen inbred popcorn lines with different Al sensitivity^24^. The inbred line 11–133 was highly resistant to Al toxicity, presenting statistical significance with the greatest RRG (0.15–0.37), no damage on root apices, lower hematoxylin staining score, and low Al accumulation (926.4 μg/g). The inbred line 11–60 was the most sensitive to Al toxicity, presenting the lowest RRG (0.02–0.06), strong hematoxylin staining, epidermal degradation, and high Al accumulation (1660.3 μg/g).

### Growth conditions

First, we treated seeds with fungicide (Captan 400) and germinated them at 25 °C ± 1 °C in a growth chamber for 7 days. Seedlings with uniform growth were picked randomly and transferred to a nutritive solution with constant aeration to acclimate for 24 h. The nutrient solution composition was 1 mM KCl, 1.5 mM NH_4_NO_3_, 1 mM CaCl_2_, 45 µM KH_2_PO_4_, 200 µM MgSO_4_, 500 µM Mg(NO_3_)_2_, 155 µM MgCl_2_, 11.8 µM MnCl_2_·4H_2_O, 33 µM H_3_BO_3_, 3.06 µM ZnSO_4_·7H_2_O, 0.8 µM CuSO_4_.5H_2_O, 1.07 µM Na_2_MoO_4_·H_2_O, and 77 µM Fe-EDTA^56,57^. Then, the treatment group was subjected to aluminum stress with 540 µM of AlCl_3_ (160 μM Al^3+^) at pH 4.5 for 72 h. The seedlings were maintained in a growth chamber at 25 °C with a 12/12-h light/dark cycle. Roots from three biological replicates were collected and immediately frozen in liquid nitrogen.

### RNA isolation and transcriptome sequencing

RNA from roots was isolated with Trizol LS reagent (Invitrogen, USA) according to the manufacturer’s protocol. The RNA was treated with DNase I Amp Grade (Thermo Scientific) to remove contaminated DNA and then quantified by spectrophotometry (NanoDrop 2000c, Thermo Scientific). The RNA integrity was verified by electrophoresis on 1.6% agarose gel in the presence of ethidium bromide. After quantification, the RNA samples were sent to Macrogen Inc. (Seoul, South Korea), where the libraries were generated using the TruSeq Stranded mRNA kit and sequenced using the Illumina HiSeq 2500 platform.

### Read preprocessing and differential expression analysis

Quality control was first performed using the FastQC program (version 0.11.8)^58^ to check the sequencing quality and identify reads with adapter contamination. Then, the raw reads were trimmed and filtered, and adapters were removed using Trimmomatic (version 0.38)^59^. The clean reads of all 12 samples were aligned to the maize reference genome (B73 RefGenv4) using Bowtie2 (version 2.3.3.1)^60^ and TopHat (version 2.1.1)^61^ with default settings for all parameters. The Cuffdiff (v2.2.1)^62^ program was used with default parameters to calculate gene expression levels and to identify DEGs in terms of fragments per kilobase per million mapped reads (FPKM). We considered DEGs showing FDR < 0.01 and a log2 fold change value (treated/control) > 1 as up-regulated genes and those with values < − 1 as down-regulated genes. To assess the line groups, PCA was conducted using the *stats* (version 3.4.4) R-package and plotted in Prism 5 (GraphPad).

### Gene function annotation and pathway analysis

Functional enrichment of all DEGs in both lines was conducted using OmicsBox (version 1.2.4) (http://biobam.com/omicsbox). Protein sequences from each DEG were subjected to a similarity search against the UniRef Enriched KEGG Orthology (UEKO) database (http://maxixe.icb.ufmg.br/ueko/) using BLAST (version 2.7.1)^63^. A script was developed to parse the output and return the KO identifier from each corresponding gene in both lines. The pathway analysis was carried out via Interactive Pathways Explorer (iPath) (version 3) (https://pathways.embl.de/) using the KO identifier. Heatmaps were produced using *heatmaply* (version 1.0.0)^64^ R-package.

### Co-expression network

The DEGs from each line were used to construct an interaction network. The first-degree of interaction was retrieved from STRING (version 10.5) (https://string-db.org). The minimum required interaction score set was 0.7, and the selected active interaction source was “co‐expression.” The resulting protein–protein interaction network was used as an input for downstream analysis in Cytoscape (version 3.7.1).

### Real-time qPCR

RNA from three independent replicates from each treatment was treated with DNase I Amplification Grade (Invitrogen, USA), and the cDNA was synthesized from 2 µg of RNA using the SuperScript Reverse Transcriptase kit (Invitrogen, USA). Real-time qPCR for nine genes identified as differentially expressed in at least one of the inbred lines was performed with an ABI 7500 (Applied Biosystems, USA). The primers were designed using Primer Express software (Applied Biosystems, USA), and the specificity was confirmed by BLAST in the Phytozome database (Supplementary Table S5).

The real-time qPCR reactions were performed using 1 μL of cDNA diluted to 1:10, 5 μL of forward and reverse primers mixed at 1.5 μM (each primer), and 6 μL of SYBR Green PCR Master Mix. The experiment was conducted using three biological replicates for each genotype (different samples from the ones used for the RNA-Seq experiment) and two technical replicates. The maize 18S rRNA was used as an endogenous control: 18S-Fw: GACTACGTCCCTGCCCTTTG and Rev-18S: TCACCGGACCATTCAATCG. The relative expression was estimated using the 2^−ΔCt^ method. The results and the statistical analysis were plotted using GraphPad Prism.

### Consent to participate

All authors consented to participate of this research.

### Declaration of use of plant material

The popcorn seeds used in this article followed the national standards required by Ministry of Agriculture, Livestock and Supply (MAPA), agency that regulates production, processing, repackaging, storage, analysis or seed trading activities in Brazil, according to Decree Nº. 10.586, of December 18, 2020, which regulates Law Nº. 10.711, of August 5, 2003. We emphasize that none of the seeds were collected for this work, once they belong to the Germplasm Bank of UFV and come from several cycles of interpopulation recurrent selection and more recently have been evaluated for some abiotic stresses by the Popcorn Breeding Program of UFV and that all works have the institution's full consent for its realization.

## Supplementary Information

Supplementary Information.

## Data Availability

The data sets supporting the results of this article are available in the NCBI SRA repository, http://www.ncbi.nlm.nih.gov/bioproject/508768.
