# Supplementary material for: Uncovering the transcriptional response of popcorn (Zea mays L. var. everta) under long-term aluminum toxicity
Source: Sci Rep. 2021 Oct 4;11:19644. doi: 10.1038/s41598-021-99097-z (PMC8490451; doi:10.1038/s41598-021-99097-z)
Supplement: Supplementary file 1 — Supplementary Information. [file 41598_2021_99097_MOESM1_ESM.zip › Supplementary Figure S1.pdf]

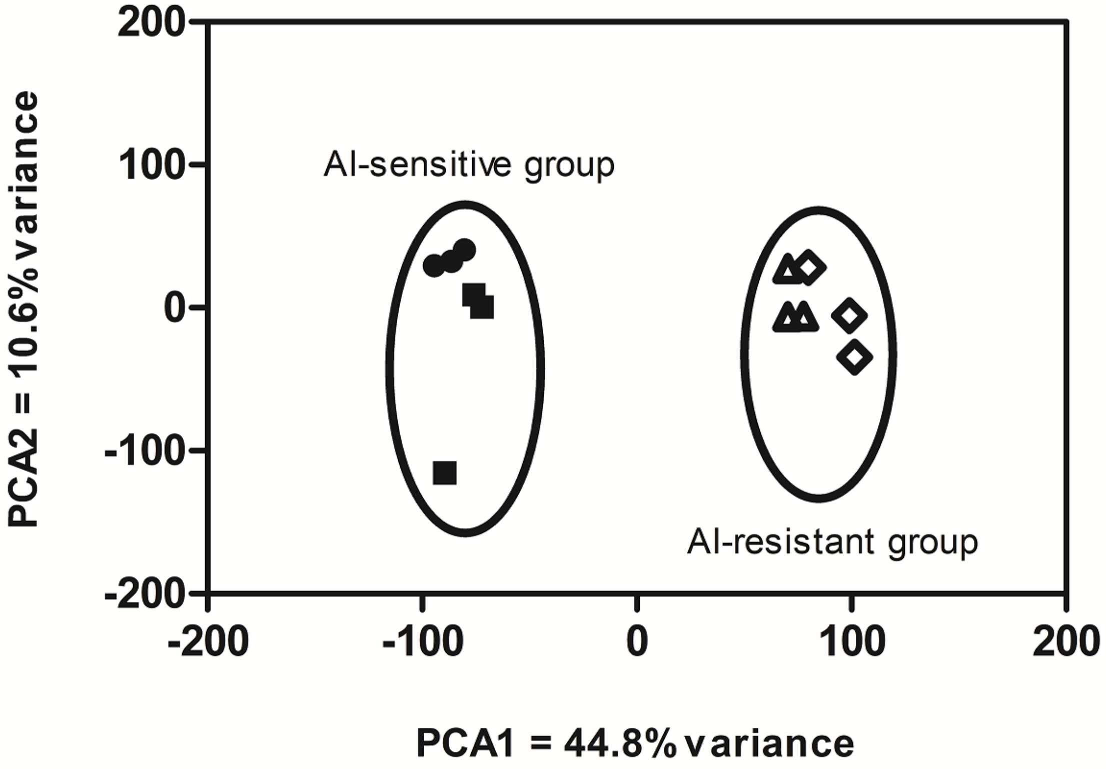

Supplementary Figure S1 PCA plot analysis using FPKM values and performed in stats R package and plotted with Prism 5 (●: Al-sensitive control; ■: Al-sensitive treatment; Δ: Al-resistant control; ◊: Al-resistant treatment).
