# Supplementary material for: Uncovering the transcriptional response of popcorn (Zea mays L. var. everta) under long-term aluminum toxicity
Source: Sci Rep. 2021 Oct 4;11:19644. doi: 10.1038/s41598-021-99097-z (PMC8490451; doi:10.1038/s41598-021-99097-z)
Supplement: Supplementary file 1 — Supplementary Information. [file 41598_2021_99097_MOESM1_ESM.zip › Supplementary Figure S4.pdf]

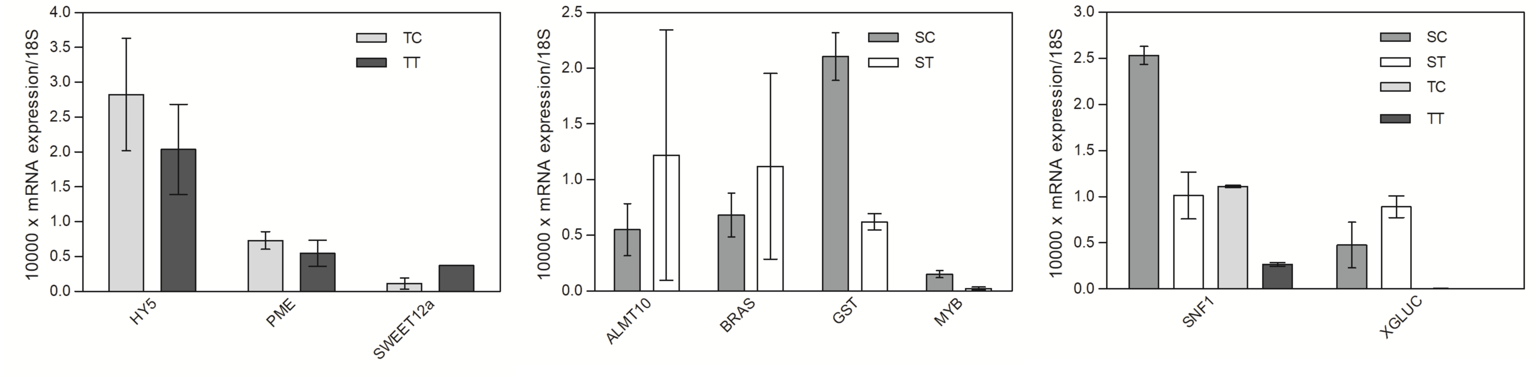

Supplementary Figure S4 DEGs identified by RNA-seq were validated by RT-qPCR. The gene expression was calculated using the $2^{-\Delta Ct}$ method and 18S as the endogenous control. The results are from three independent biological replicates and two technical replicates.

SC: Al-sensitive control; ST: Al-sensitive treatment; TC: Al-resistant control; TT: Al-resistant treatment.
