# Supplementary material for: Uncovering the transcriptional response of popcorn (Zea mays L. var. everta) under long-term aluminum toxicity
Source: Sci Rep. 2021 Oct 4;11:19644. doi: 10.1038/s41598-021-99097-z (PMC8490451; doi:10.1038/s41598-021-99097-z)
Supplement: Supplementary file 1 — Supplementary Information. [file Supplementary Figure S2.pdf › Supplementary Figure S2.pdf]

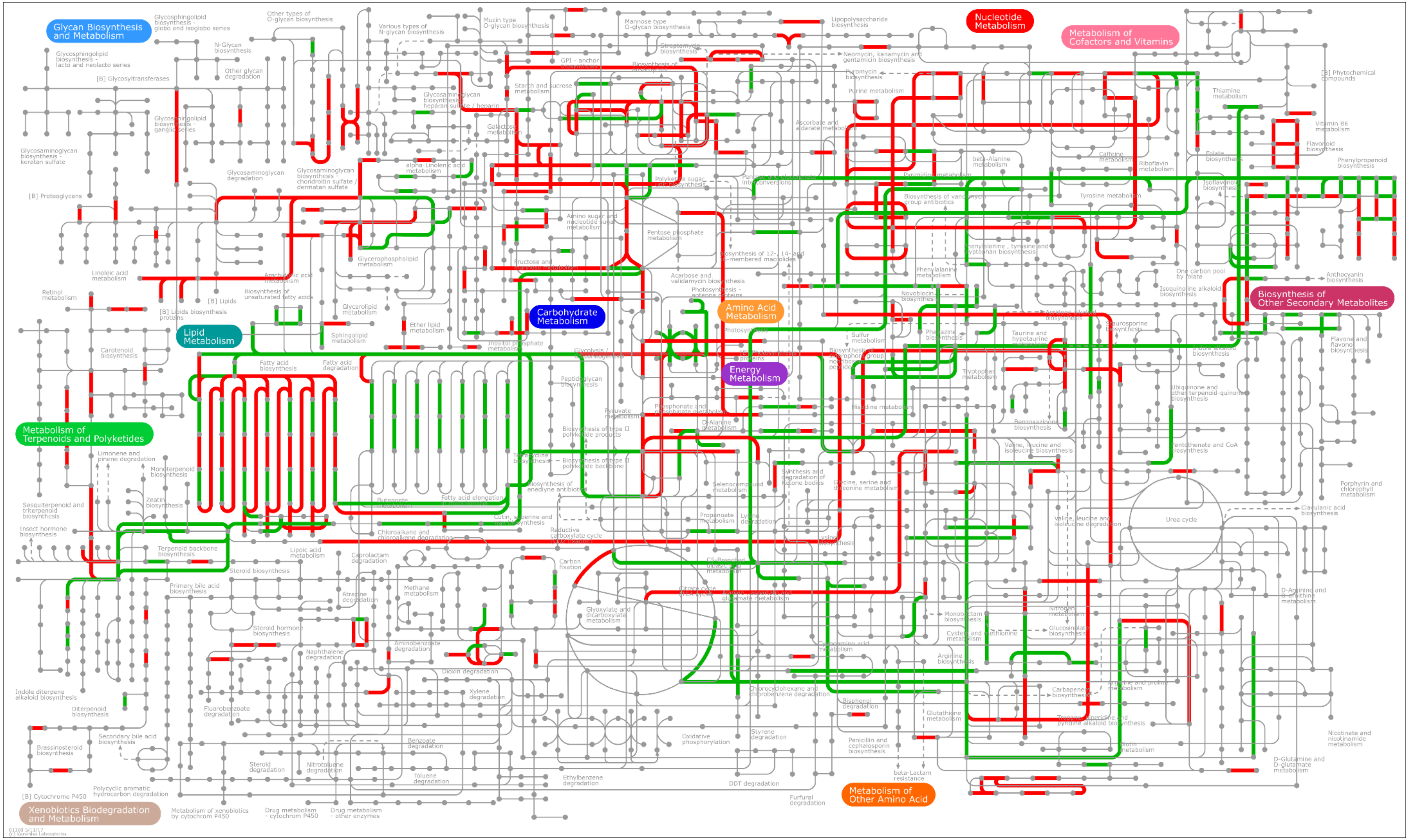

Supplementary Figure S2 Interactive Pathways Explorer analysis in Al-resistant. Red lines indicate activated pathways and green lines indicate deactivated pathways.
